# Advantages of systematic trunk SPECT/CT to planar bone scan (PBS) in more than 300 patients with breast or prostate cancer

**DOI:** 10.18632/oncotarget.25860

**Published:** 2018-08-03

**Authors:** Vincent Fleury, Ludovic Ferrer, Mathilde Colombié, Daniéla Rusu, Maëlle Le Thiec, Françoise Kraeber-Bodéré, Loïc Campion, Caroline Rousseau

**Affiliations:** ^1^ Nuclear Medicine Unit, ICO Cancer Center, Saint Herblain, France; ^2^ Nantes-Angers Cancer Research Center, INSERM U892, CNRS UMR 6299, University of Nantes, Nantes, France; ^3^ Biometrics Unit, ICO Gauducheau Cancer Center, Saint Herblain, France; ^4^ Medical Physics Unit, ICO Cancer Center, Saint Herblain, France

**Keywords:** SPECT/CT, bone scan, bone metastases, bone index, prostate cancer

## Abstract

**Propose:**

The aim of our study was to evaluate the potential benefit of a systematic trunk SPECT/CT associated with a Planar Bone Scan (PBS) in breast cancer (BC) and prostate cancer (PCa) patients at initial staging or recurrence.

**Results:**

In 328 patients, sensitivities and specificities were between 74.4–93% and 78.8–97.5% for PBS and 97.7–100% and 96.8–98.6% for SPECT/CT respectively. PBS was considered equivocal for 67 compared to only 6 patients for trunk SPECT/CT. Regardless of “optimistic” or “pessimistic” analysis of equivocal trunk SPECT/CT lesions, the trunk SPECT/CT was almost perfect, allowing to rely on this result for excluding metastatic disease which was corroborated by ROC curve analysis. The trunk SPECT/CT allowed downstaging for 62 patients (19%) and upstaging for 11 patients.

**Materials and Methods:**

PBS and a trunk SPECT/CT were systematically performed in all patients. Independent review of PBS and trunk SPECT/CT was performed for each patient and an abnormality interpretative score (Sc) with 3 levels was built: Sc 1: metastatic or probably metastatic pattern, Sc 2: equivocal pattern, Sc 3: benign or probably benign pattern or no abnormality. The bone pattern status was defined by at least 1 year follow-up. The clinical impact was evaluated in terms of down and upstaging in patient analysis.

**Conclusions:**

Trunk SPECT/CT improves the performance of PBS in BC and PCa assessments and results in improvements in both the detection performance of bone metastases as well as a better characterization of equivocal lesions.

## INTRODUCTION

Breast cancer (BC) and prostate cancer (PCa) are very common neoplasms with metastatic progression in 30% of cases [1, 2].

Currently, Planar Bone Scan (PBS) is recommended in staging of metastatic high-risk PCa [3, 4] and in BC with adverse prognostic factors [5]. However, specificity is limited, with many cases of false positive results [6]. Technological innovations have made it possible to improve accuracy, first with single-photon emission tomography (SPECT) [7–9] and then with the advent of hybrid cameras enabling SPECT to be coupled with CT. SPECT/CT allows a better characterization of uptake foci, thus improving the specificity and decreasing the number of equivocal lesions that do not contribute to diagnosis [10–15]. This improvement in the performance of bone scintigraphy has, due to a more exhaustive assessment, improved the therapeutic strategy in these patients [16, 17]. However, data in the literature shows that SPECT/CT is not systematically performed and is usually targeted by whole body scan and focus on increased radiotracer uptake. The potential benefit of carrying out a SPECT/CT, systematically associated with PBS and exploring the whole axial skeleton, would be to improve the sensitivity and especially the specificity of focal bone lesions. Whilst this has been discussed in the literature, the results differ depending on the studies [9, 18, 19].

The aim of our study was to evaluate the potential benefit of a trunk SPECT/CT systematically focused on the axial skeleton in addition to PBS in BC and PCa patients at initial staging or with a suspicion of recurrence.

## RESULTS

### Study population

**Figure 1:**
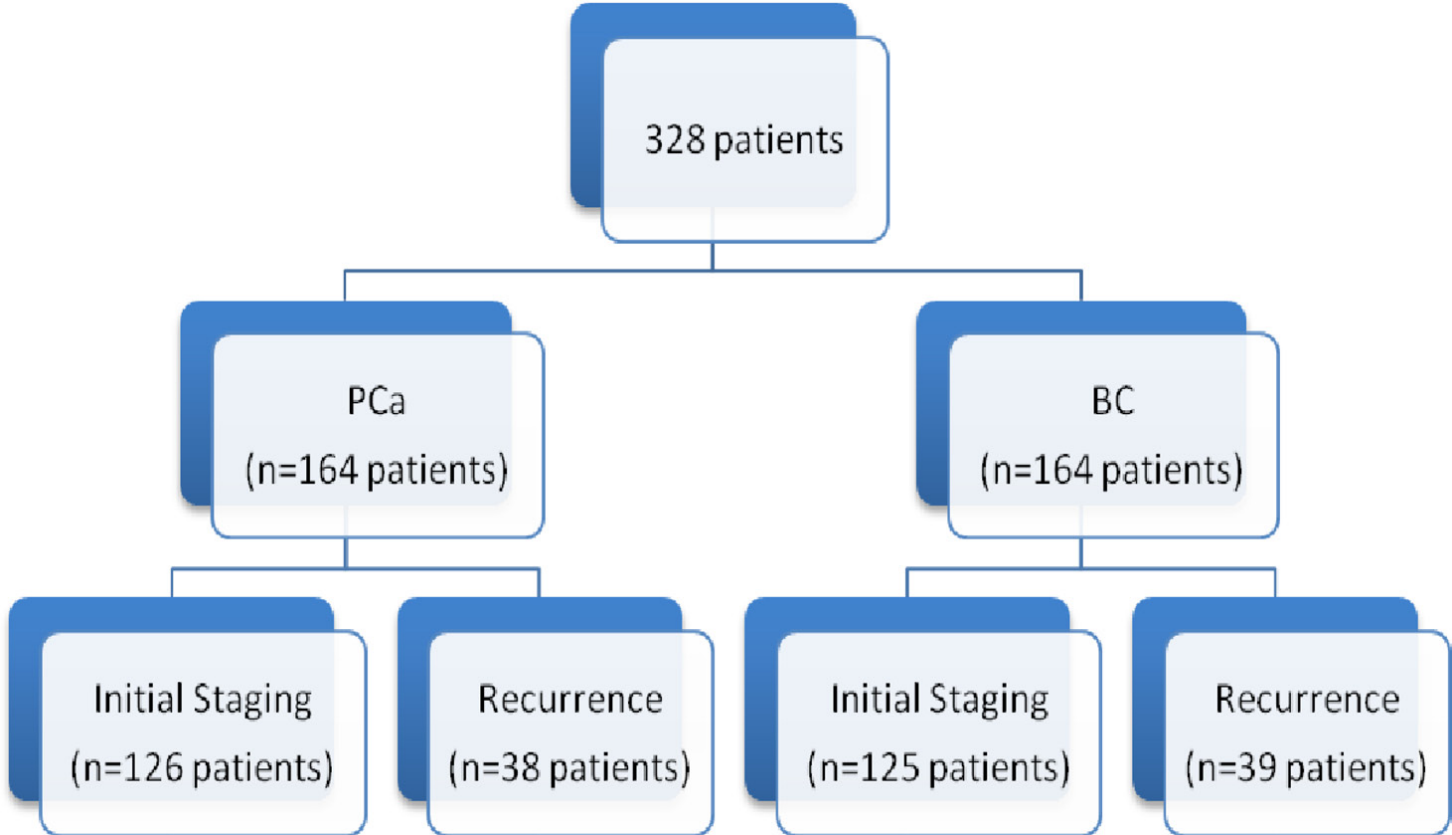
Patients distribution examined by PBS and SPECT/CT

Between January to October 2015, 328 consecutive patients were retrospectively included in this study. An equal partition was chosen between the 2 pathologies (Figure 1). Patient characteristics are summarized in Table 1.

**Table 1:** Characteristics of prostate and breast cancer patients

Prostate cancer patients		Breast cancer patients	
**Initial staging**	**126 patients**	**Initial staging**	**125 patients**
Age	69 (49–88)	Age	53 (32–90)
PSA	10 (2.42–641)	Stage :	
Gleason score		I	30 (24%)
6	12 (9.5%)	IIa	38 (30,4%)
7	77 (61.1%)	IIb	31 (24,8%)
8	26 (20.6%)	III	26 (20,8%)
9	10 (12.6%)	SBR score :	
Undetermined	1 (0.01%)	1	9 (7,2%)
D'amico Classification		2	58 (46,4%)
Intermediate risk	75 (59.5%)	3	55 (44%)
High risk	51 (40.5%)		
**Recurrence**	**38 patients**	**Recurrence**	**39 patients**
Age	76 (50–92)	Age	62 (36–86)
PSA	7.995 (0.3–484)		

**Figure 2:**
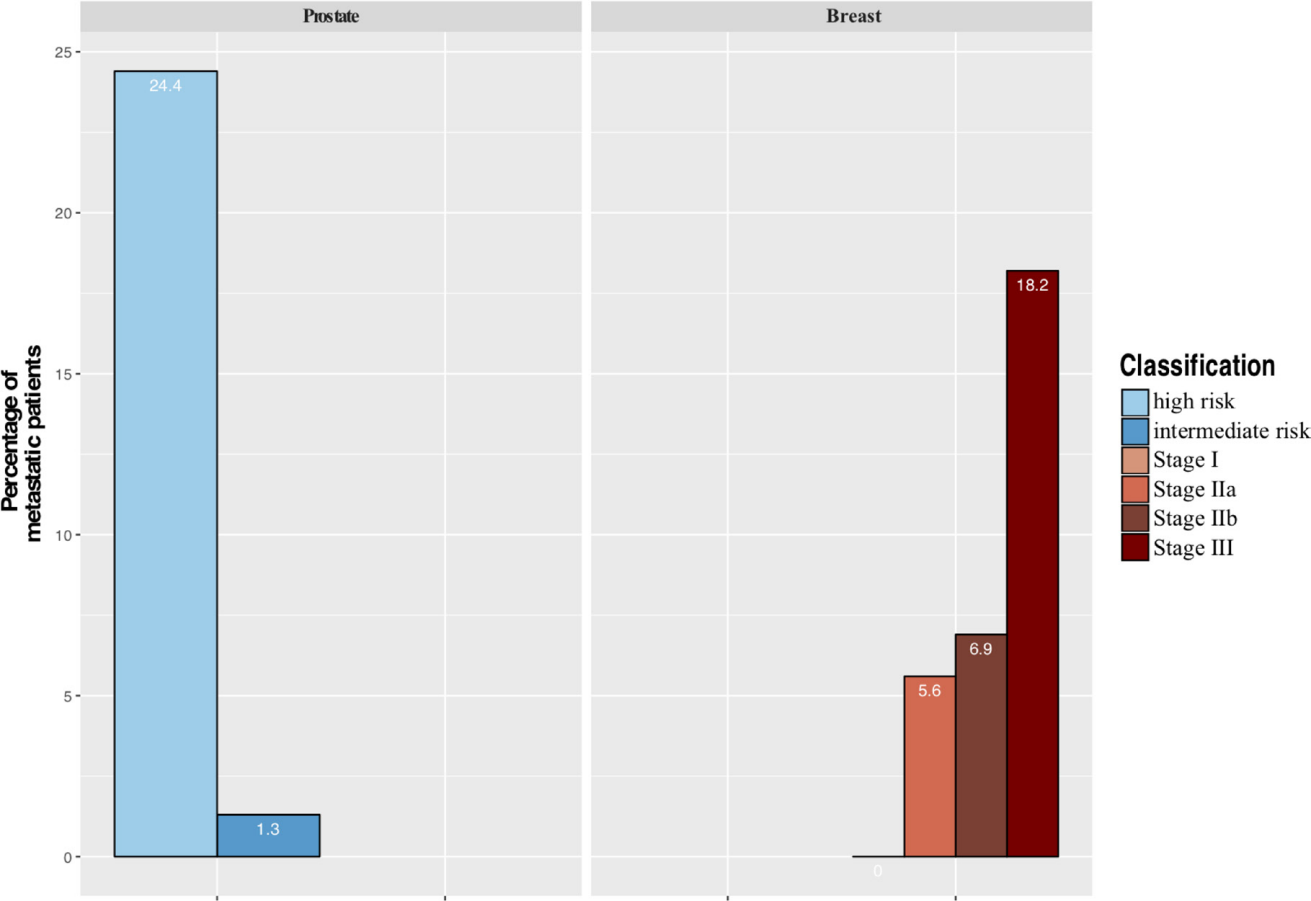
Distribution of metastatic patients at baseline by stage AJCC for breast cancer and d'Amico classification for prostate cancer

The majority of patients corresponded to AJCC stage III BC patients and high risk of D' Amico classification PCa patients (Figure 2).

A total of 42 patients were bone metastatic (12.8%), 19 at initial and 23 at suspected recurrence. This diagnosis was confirmed during follow-up, histological examinations for 2 patients, imaging for 15 patients and clinical follow-up for 25 patients. Bone division (7 parts for PBS and 5 for trunk SPECT/CT) allowed to verify that the lesions metastatic status that was obtained at follow-up, corresponded to the suspicious site on the image (ie there was concordance) and was not due to disease progression.

### Comparison of PBS and trunk SPECT/CT

**Figure 3:**
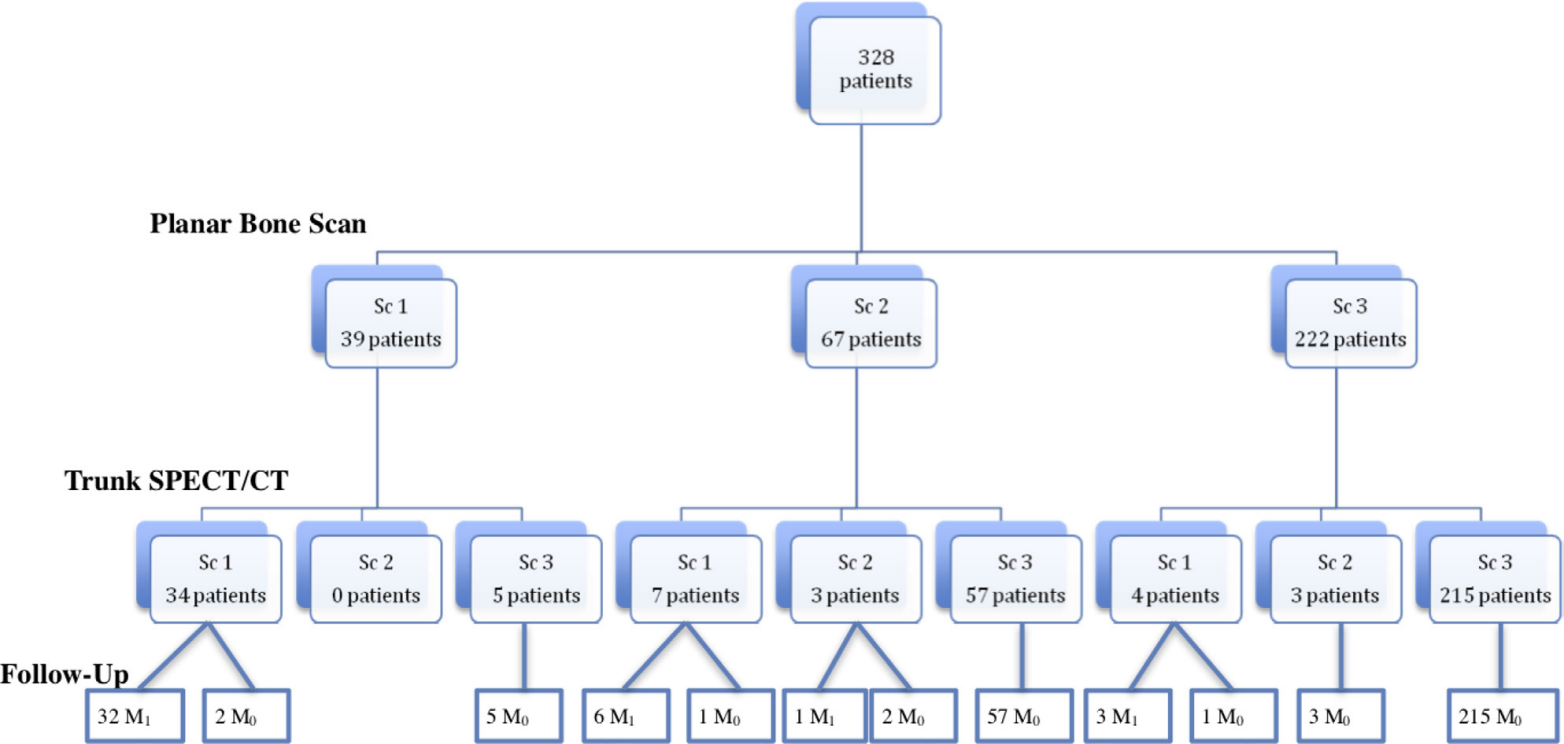
General population diagram according to PBS and trunk SPECT/CT results

PBS results on one hand, and trunk SPECT/CT results on the other, were compared to the follow-up results as summarized in Figure 3. The analysis of the PBS acquisition was in favour of metastatic lesions (Sc 1) for 39 patients (11.9%) compared to 45 (13.7%) with trunk SPECT/CT. Furthermore, of these 41 patients, metastatic status was confirmed in 39 patients (95.1%). Among the 39 PBS patients (Sc 1), trunk SPECT/CT was not considered metastatic in 5 patients whereas for 2 patients, the PBS showed multiple regions of suspect uptake in more than 3 different anatomical regions. Comparison of the different regions of bone by PBS and trunk SPECT/CT allowed us to identify 3 patients with a lesion scored as metastatic on trunk SPECT/CT and detected outside the PBS incriminated region, which would have avoided a targeted SPECT/CT by the location of the PBS indeterminate lesion.

PBS was considered equivocal in 67/328 patients (20.4%). Trunk SPECT/CT was in favour of benign uptake for 57 of these patients (85%), which was confirmed by the follow-up. Trunk SPECT/CT identified suspicious lesions in 7 patients, 6 of whom were confirmed as metastatic. In 3 of these patients, regions of suspect uptake identified by trunk SPECT/CT was detected in a different anatomical region from that identified by PBS. Examination remained equivocal in only 3 patients (4.5%) after the integration of trunk SPECT/CT data, of which one was metastatic.

**Figure 4:**
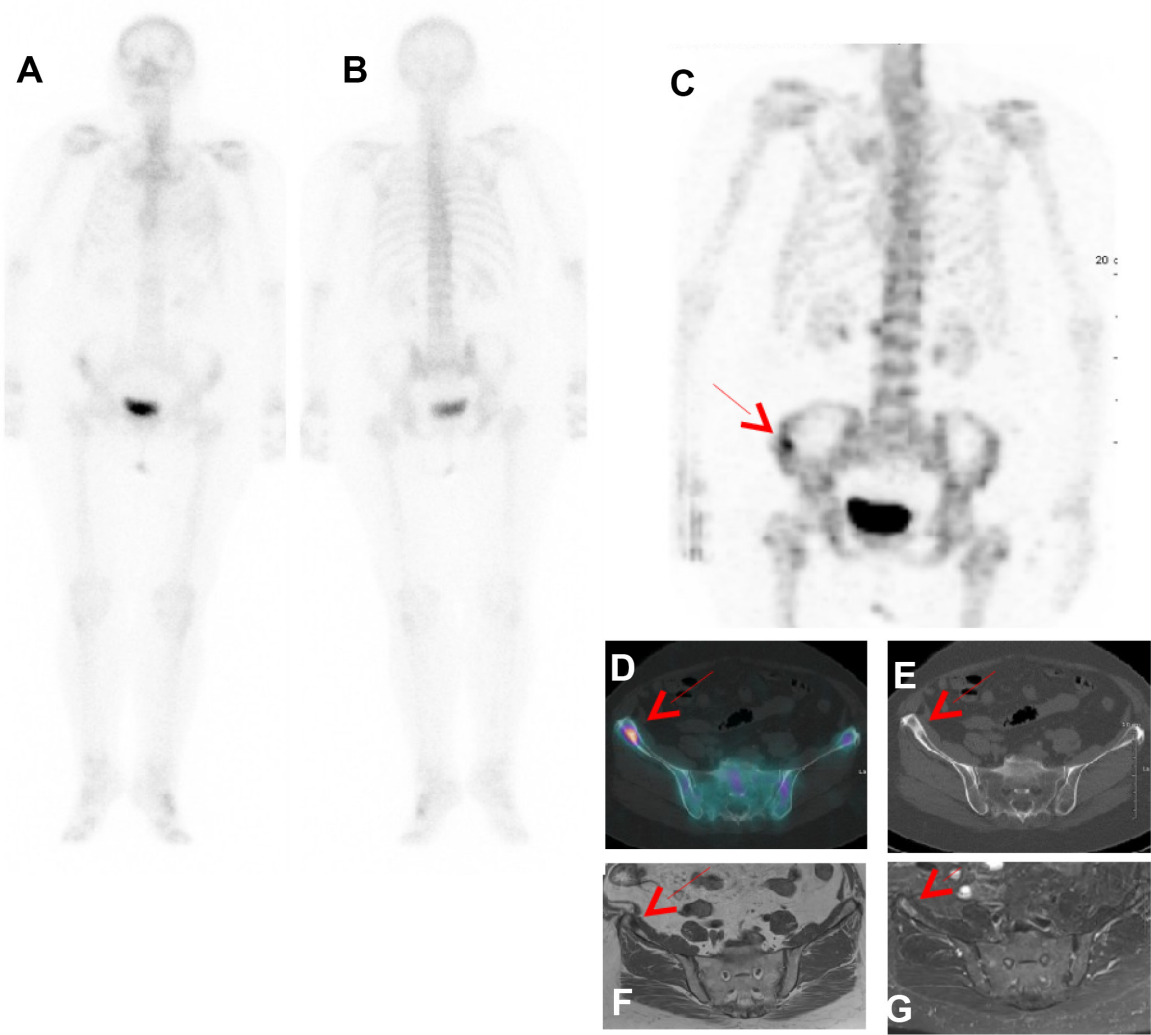
Patient with PBS Sc 3 and trunk SPECT/CT Sc 1 proved as true positive by follow-up In a 79-year-old female with newly diagnosed breast cancer with involved lymph node (T1N1), PBS in ventral (**A**) and dorsal (**B**) projections did not reveal any suspicious bone lesio. Right iliac aisle uptake (red Arrow) was seen on (**C**) trunk SPECT/CT MIP (Maximal Intensity Projection) and (**D**) fusion SPECT/CT with a sclerosis lesion observed on CT (**E**). Bone metastasis was confirmed by MRI respectively T1 and T1 gadolinium Fat sat (**F**, **G**) and clinical follow up.

Among the 222 patients with a PBS score of 3 (benign, probably benign or absence of lesion), trunk SPECT/CT identified a confirmed metastatic bone lesions in 3 patients, as shown in Figure 4, and one case of false positive in a patient with lumbar uptake. On the other hand, trunk SPECT/CT incorrectly identified a suspected lesion in 3 patients.

Trunk SPECT/CT has drastically reduced the number of equivocal uptake to only 6 patients (1.8%) compared to 67 patients (20.4%) for PBS.

In summary, the SPECT/CT trunk scans allowed downstaging for 62 patients (19%) and upstaging for 11 patients, 4 of whom had no abnormalities on the PBS and 7 of whom had an equivocal lesion on the PBS within a different region than the one selected as pathological on the trunk SPECT/CT.

**Figure 5:**
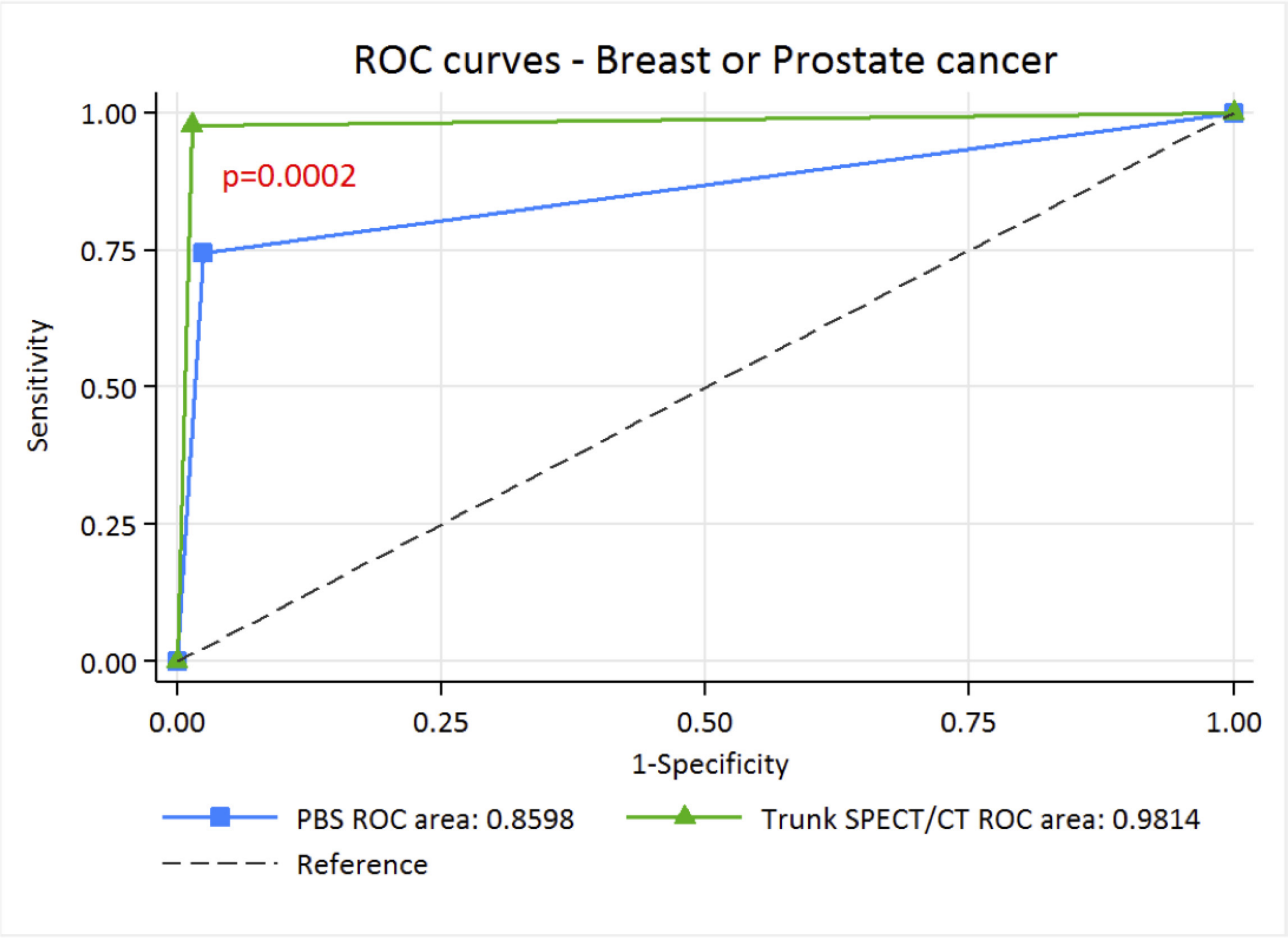
ROC curve of PBS and trunk SPECT/CT for “optimistic” analysis in breast and prostate cancer patients

**Figure 6:**
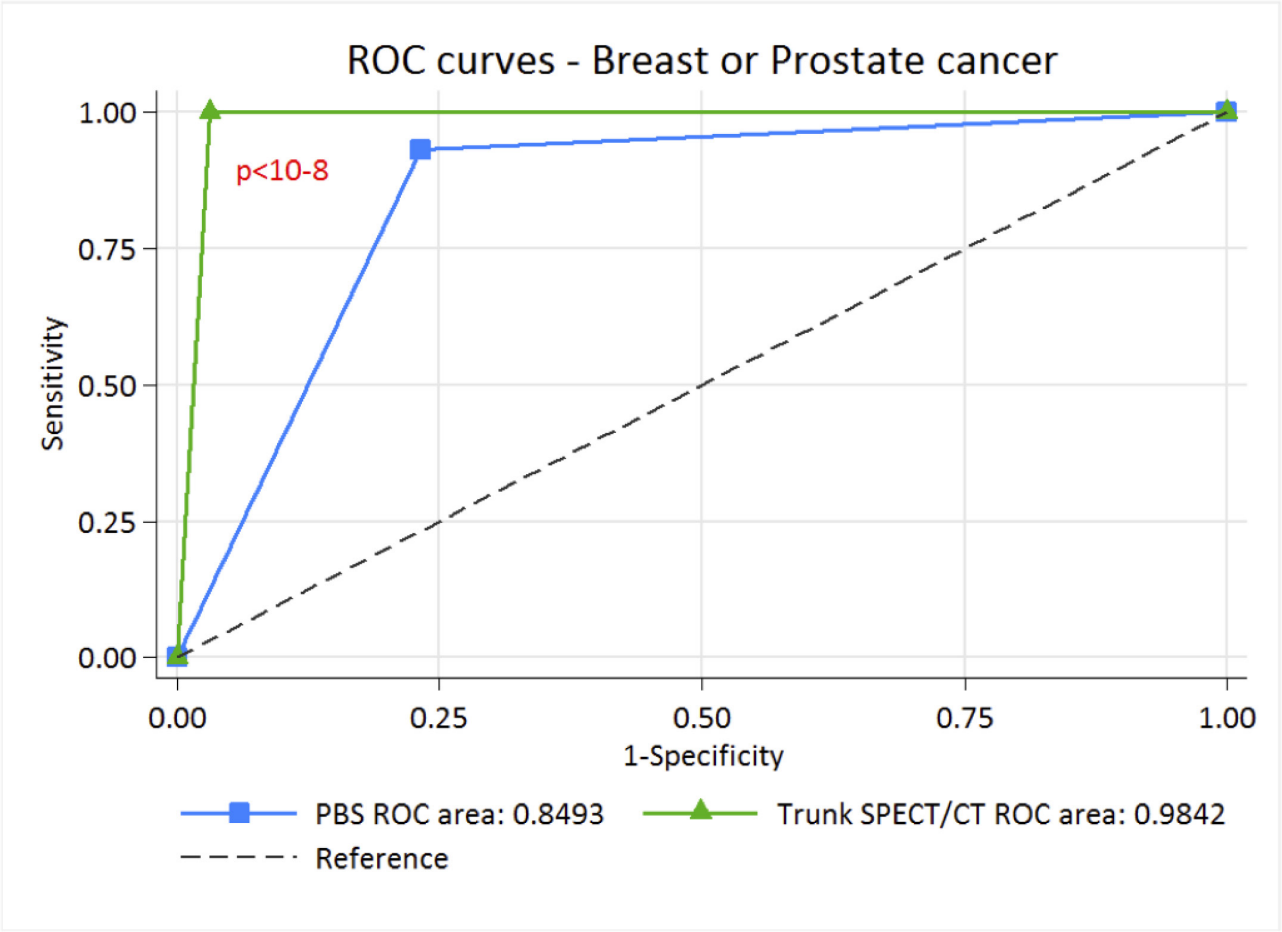
ROC curve of PBS and trunk SPECT/CT for “pessimistic” analysis in breast and prostate cancer patients

Depending on whether the lesions were considered benign for “optimistic analysis” or metastatic for “pessimistic analysis”, sensitivities and specificities were between 74.4–93% and 78.8–97.5% for PBS and 97.7–100% and 96.8–98.6% for SPECT/CT, the NPV of trunk SPECT/CT was almost perfect, allowing this result to be used to exclude metastatic bone lesions (Tables 2 and 3). This is corroborated by Receiver Operating Characteristic (ROC) curve analysis, where with “optimistic” analysis, AUC is higher for trunk SPECT/CT with 0.9814 [IC 95%: 0.95756–1.00000] compared to 0.8598 [IC 95%: 0.79322–0.92640] for PBS *p <* 0.0002) (Figure 5). Similarly, with the “pessimistic” analysis, AUC of trunk SPECT/CT is higher (AUC 0.9842 [IC 95%: 0.974–0.994] compared to 0.8493 [IC 95%: 0.803–0.895] for PBS, *p <* 10–6) (Figure 6). However, there was no significant difference between PBS AUCs according to the “pessimistic” and “optimistic” analyses (*p* = 0.74) and trunk SPECT/CT AUCs according to the same analyses (*p* = 0.81).

**Table 2:** “Pessimistic analysis” of equivocal examination retained as metastatic

Parameters	PBS	Trunk SPECT/CT	*p*
**Sensitivity**	93% (90.3–95.8)	100% (100–100)	0.21
**Specificity**	76.8% (72.3–81.4)	96.8% (94.9–98.7)	<10^−6^
**PPV**	37.7 (32.4–43)	82.7% (78.6–86.8)	<10^−6^
**NPV**	98.7% (97.4–99.9)	100% (100–100)	0.088

Sensitivity, specificity, positive predictive value (PPV), negative predictive value (NPV) of PBS and trunk SPECT/CT for the diagnosis of bone metastases in breast and prostate cancer patients.

**Table 3:** “Optimistic analysis” of equivocal examinations retained as benign uptake

Parameters	PBS	Trunk SPECT/CT	*p*
**Sensitivity**	74.4% (69.7–79.1)	97.7% (96–99.3)	0.003
**Specificity**	97.5% (95.9–99.2)	98.6% (97.3–99.9)	0.545
**PPV**	82% (77.9–86.2)	91.3% (88.2–94.3)	0.331
**NPV**	96.2% (94.1–98.3)	99.6 (94.1–98.2)	0.006

Sensitivity, specificity, positive predictive value (PPV), negative predictive value (NPV) of PBS and trunk SPECT/CT for the diagnosis of bone metastases in breast and prostate cancer patients.

Finally, the systematic addition of trunk SPECT/CT to PBS resulted in an additional radiation exposure of 6.25 ± 1.79 mSv due to the low-dose CT.

## DISCUSSION

To our knowledge, this is the largest study evaluating the performance of systematically adding trunk SPECT/CT to PBS for the detection of bony metastases in BC and PCa patients.

The major point of this study is the excellent performance of trunk SPECT/CT compared to PBS with an AUC of 0.98 in ROC analysis, a result higher than that observed in a similar study conducted by Palmedo. *et al.* [18]. These results imply a significant benefit can be achieved in systematically performing trunk SPECT/CT for any patient undergoing initial staging or suspected relapse for prostate or breast cancer in addition to PBS. How does our study improve on the findings published by Palmedo *et al.*? Our results confirm the superior performance of trunk SPECT/CT over PBS with external validation on the same target population, which is required as a necessary step before being implemented into routine clinical practice [20]. The overall imaging time remains quite acceptable (25 min) for the patient due to a time saving by acquiring the PBS with a fast whole body scan combined with a non-linear spatial resolution restoration filter. Additional irradiation due to Low Dose CT with morphologically modulated low dose CT for trunk SPECT/CT can be considered acceptable with an effective dose of around 6 mSv. Thus, a significant improvement in diagnostic accuracy and patient management can be achieved without substantially increasing the radiation dose.

The sensitivity and specificity of trunk SPECT/CT were very high in our study but similar to previous studies on a per patient analysis [18, 21]. For PBS, our results in terms of sensitivity and specificity are also significantly better than those published by Jambor *et al.* and Lofgren *et al.*, where they compared PBS to MRI or FNa PET/CT, and performed a whole-body scan much slower than ours (10 to 13 cm/min, compared to 30 cm/min for our study). Under these conditions, the overall imaging time was 50–60 min in the study of Löfgren *et al.* and more than 35 min for the study of Jambor *et al.* [22, 23], which could be considered especially uncomfortable for a patient with bone metastases. While the performance of trunk SPECT/CT was similar in cases of “optimistic” or “pessimistic” analysis of equivocal data, our result with the PBS were less robust, and reflects the difficulty of characterizing certain uptake in planar imaging. This diagnostic outcome may require additional imaging, along with associated costs, time and impact on the patient. Our study confirms the excellent performance of trunk SPECT/CT for the reliable characterization of equivocal lesions in PBS, since for 64/67 patients (95.5%) these anomalies were correctly characterized. These results are higher than in other studies where SPECT/CT remained equivocal in 8 to 14% of patients [10–12]. To improve the detection of bone metastases, it would seem appropriate to examine the bone marrow hematopoietic located within the axial skeleton as thoroughly as possible, because it is the main site of bone metastatic dissemination. However, there is as yet no specific guidelines on the bone scan protocol. Some people recommend systematic trunk SPECT/CT after PBS [18, 22]. Indeed, a study has shown a higher sensitivity of WB SPECT/CT compared to targeted SPECT/CT, allowing a drastic modification of staging [21]. For others, trunk SPECT/CT has no added value compared to a targeted SPECT/CT [19]. Although SPECT/CT has better accuracy than SPECT alone some authors are considering the use of WB SPECT instead of a standard PBS to avoid additional radiation exposure due to CT without compromising examination performance [24].

In our study, trunk SPECT/CT allowed for upstaging in 11 of our patients. On their PBS, 4 of them had no lesion. For these, the SPECT/CT could not be guided. The other 7 patients had at least one indeterminate lesion. In 3 of these 7 patients, a suspicious lesion on the trunk SPECT/CT was detected outside the PBS incriminated region, which would have escaped a targeted SPECT/CT by the location of the PBS indeterminate lesion. Whilst the usefulness of systematic trunk SPECT/CT has been discussed in the literature, there are as yet no specific guidelines concerning optimal imaging protocols. More recently, a study showed a higher sensitivity of trunk SPECT/CT compared to targeted SPECT/CT as well as a drastic modification of staging [21], in contrast to the findings of others [19].

The “standard of reference” of our study was based on a clinical follow-up of at least one year, sometimes combined, according to the wishes of the clinics. Histological confirmation was requested for only 2 patients. False negative test results could be evoked. However, the clinical follow-up, longer than 1 year, almost rules out undetected bone metastasis, particularly in case of a negative examination. Given this “standard of reference”, we decided not to carry out an analysis by region but only an analysis by patient. Indeed, it is ethically impossible to obtain histological proof for every lesion and further imaging confirmation seems illusory when the number of lesions per region becomes large.

## MATERIALS AND METHODS

In our institution, a trunk SPECT/CT in addition to whole-body scintigraphy (WBS) is systematically performed for BC staging according to the guidelines of the ESMO [5] and intermediate and high risk PCa according to the D'amico classification, or in cases of suspicion of recurrence. All patients received written information and we obtained consent allowing the use of their clinical data for research purposes under a protocol approved by the ethics committee.

### Image acquisition

The acquisition of whole body images on the anterior and posterior views was started 3 hours after IV injection of 9 MBq/kg of 99mTc-HMDP (Osteocis, IBA Cis Bio), with 2 types of gamma cameras (Discovery NMCT670, GE Healthcare, USA, and Symbia T2, Siemens, Germany), equipped with low energy-high resolution collimators. The scan speed was 30 cm/min with a 256 × 1024 image matrix and energy window 140 keV ± 15%. A non-linear spatial resolution restoration filter, provided by the manufacturers, was systematically applied to obtain a PBS in 5–6 minutes.

Immediately after the PBS, a SPECT acquisition from the base of the skull to the midthighs was performed (with arms along the torso). 90 projections were acquired (10 seconds per step), followed by a 3D reconstruction with ordered subset expectation maximization (OSEM) (4 iterations and 8 subgroups) for a 128 × 128 matrix.

CT imaging was performed to attenuation correction of the SPECT and the anatomical location of the scan data. A low-dose CT was performed with the following parameters on the Symbia T2 (Siemens): modulation of mAs according to morphology (Care4D), 130 kV, slice thickness 5 mm and pitch 2. For the Discovery NMCT670 (GE Healthcare) Discovery gamma-camera, the parameters were: mA modulation (smart mA), 140 kV, slice thickness 2.5mm with a pitch of 1.375.

The overall imaging time (PBS+trunk SPECT/CT) was approximately 25 minutes.

### Data analysis and interpretation

The PBS and SPECT/CT images were assessed independently of each other by a nuclear physician specializing in oncology and osteoarticular imaging. The specialist was blinded from the patients’ clinical data, except for the type of neoplasia.

For the analysis of PBS, the skeleton was divided into 7 distinct regions: the skull, the spine segmented into 3 parts (cervical, thoracic and lumbar), the pelvis, the ribs associated with the sternum, and the appendicular skeleton. We formulated a score (Sc) at 3 levels, taking into account the uptake intensity, their number and their topography:

Sc 1: metastatic or probably metastatic lesion

Sc 2: equivocal lesion

Sc 3: benign, probably benign or absence of lesion

SPECT/CT images of all patients were read and ranked according to the same score but incorporating bone CT data. As the skull and appendicular skeleton were not in the field of view of the trunk SPECT/CT the skeleton was divided into only 5 regions: cervical, dorsal and lumbar spine, pelvis and sternum associated with ribs). Anatomical CT images of SPECT/CT were used to categorize uptake as benign and probably benign, or metastatic or probably metastatic due to corresponding morphologic findings. Typical benign lesions according to CT data were bone cysts, degenerative lesions (e.g. around joints), and fractures. When the tracer uptake was localized to osteoblastic, osteolytic or mixed lesions, the lesion was marked as metastatic or probably metastatic based on SPECT/CT. Lesions with tracer uptake which were not typically benign or malignant on CT were considered as equivocal.

### Standard reference

After Bone scan, a 12-month follow-up was carried out for all patients. It was based on the collection of clinical and biological patient data. Data from complementary investigations using morphological (MRI or CT) and/or functional imaging (FDG or F-Choline PET/CT if prostatic pathology) and/or histological evaluation was collected during the follow-up. Patients with Sc3 without recurrence or progression during follow-up were considered true negative. Patients with a Sc 1 confirmed by imaging or histological examination were considered to be true positive. Patients with a Sc 1 or 2 not confirmed by further investigations or without progression in follow-up were considered false positive. If follow-up examinations showed metastatic lesion, patients were considered as false negative.

### Statistics

Continuous parameters were described as median (range) and qualitative parameters as frequency (%) of their respective modalities. To assess links between qualitative parameters, a Pearson Chi-square test (or Fisher's exact test if necessary) was used. Statistical performance (sensitivity, specificity, positive and negative predictive values) of -1- PBS and -2- SPECT/CT were calculated using two thresholds: an “optimistic analysis” in which equivocal patients were considered negative for bone metastasis, and “pessimistic analysis” in which equivocal patients were considered positive for bone metastasis [22, 23]. AUC of the ROC curves were calculated and compared between both techniques by means of Delong test. All tests were two-sided with significance at *p* ≤ 0.05. All calculations were performed using SAS 9.4 (SAS Institute Inc., Cary, NC, USA) and R version 3.3.1 (Copyright (C) 2016 The R Foundation for Statistical Computing).

## CONCLUSIONS

The systematic addition of trunk SPECT/CT to PBS significantly improves the performance of PBS in breast and prostate cancer staging. The added value is reflected in the improved detection performance of bone metastases and a better characterization of equivocal lesions allowing a more exhaustive bone staging and thus a more adaptive and personalized treatment.

LF: acquisition and analysis of data.

MC, DR, MLT: acquisition and interpretation of data.

FKB: revising it for important content.

LC: analysis statistic of data.

CR: conception of the study, revising it for important content and final approval.

## Abbreviations

BC: Breast cancer
BSI: Bone Score Index
PBS: Planar Bone Scan
PCa: Prostate Cancer
Sc: Score
SPECT: Single Photon Emission Computed Tomography
SPECT/CT: Single Photon Emission Computed Tomography/ Computed tomography
ROC: Receiver operating characteristic
WBS: Whole Body Scan.
